# Habit strength as a partial explanation of the association between self-efficacy and self-management behaviors in type 2 diabetes: a multicenter cross-sectional study

**DOI:** 10.3389/fpubh.2026.1870512

**Published:** 2026-06-23

**Authors:** Yan Lin, Caihua Ye, Shuping Xing, Kaining Chen, Qiwei Zhou, Chengying Yu, Ying Zhou, Yane Yan, Wenfei Yang, Bin Li, Xinjun Jiang

**Affiliations:** 1 School of Nursing, Hainan Medical University, Haikou, Hainan, China; 2 Department of Endocrinology, Hainan General Hospital, Haikou, Hainan, China; 3 Department of Hyperbaric Oxygen, 928th Hospital of PLA Joint Logistics Support Force, Haikou, Hainan, China; 4 Department of Nursing Management, Hainan General Hospital, Haikou, Hainan, China

**Keywords:** behavioral pathway, habit strength, self-efficacy, self-management behaviors, type 2 diabetes mellitus

## Abstract

**Background:**

While individual behavior is governed by both conscious control and unconscious automatic processes, the extent to which habit strength influences the relationship between self-efficacy and self-management behaviors in type 2 diabetes mellitus (T2DM) remains insufficiently quantified.

**Aims:**

To examine the associations among habit strength, self-efficacy, and diabetes self-management behaviors, and to explore whether habit strength may help explain the association between self-efficacy and diabetes self-management behaviors among patients with T2DM.

**Methods:**

This was a multicenter, cross-sectional study. A total of 292 patients with T2DM were recruited from four hospitals in Hainan Province, China. Self-efficacy, habit strength, and self-management behaviors were assessed using validated questionnaires. Associations among variables were examined using correlation. Path analysis was conducted using SPSS with the PROCESS macro to explore whether habit strength may explain the association between self-efficacy and self-management behaviors.

**Results:**

Patients demonstrated moderate levels of diabetes self-management behaviors (mean = 34.15 ± 13.51). Self-efficacy was positively associated with both habit strength and self-management behaviors, and habit strength was also positively associated with self-management behaviors. Path analysis suggested that habit strength may partially explain the association between self-efficacy and self-management behaviors, accounting for 18.6% of the total effect.

**Conclusion:**

Habit strength is associated with diabetes self-management behaviors and may partially explains the relationship between self-efficacy to behavioral performance in individuals with T2DM. Interventions that support habit formation may facilitate the application of patients’ self-efficacy to sustained self-management behaviors. Incorporating strategies that reduce cognitive burden and promote automatic behavioral engagement may enhance the effectiveness of diabetes self-management support.

## 1 Introduction

Diabetes represents a major global health challenge and is one of the fastest-growing chronic diseases worldwide. According to data from the Non-Communicable Diseases Risk Factor Collaboration (1) and the International Diabetes Federation (2), approximately 828 million adults globally have diabetes. In China alone, about 148 million adults are affected, with over 110 million diagnosed with type 2 diabetes mellitus (T2DM). In 2021, more than 6.7 million individuals aged 20–79 died from diabetes-related complications (3). Given that substantial physical, psychological, and economic burdens imposed by T2DM on individuals and society (4, 5), effective management of this condition is a crucial global health priority.

Continuous self-management is essential for delaying or preventing complications in T2DM patients (3). Diabetes self-management behaviors refer to proactive measures undertaken by individuals to effectively control their condition (6), encompassing healthy dietary habits, regular physical activity, foot care, self-monitoring of blood glucose, and medication adherence. These behaviors, though vital, are both complex and demanding. Modifying and maintaining effective self-management behaviors is challenging, influenced by various factors, and requires sustained efforts (7). Therefore, investigating methods to enhance and sustain these behaviors in patients with T2DM is necessary.

Habitual behaviors refer to actions performed automatically, rapidly, and efficiently in response to specific cues, typically with minimal conscious effort (8, 9). Habitual behavior can be conceptualized as the psychological process underlying an action, emphasizing automaticity in both the process and outcome of the behavior (10, 11). Habit strength serves as an indicator of automaticity for evaluating self-management behaviors (12). Previous study showed that strong habits significantly contribute to sustaining health-promoting behaviors (10). Meanwhile, research suggests that behaviors and their initiation involve complex interactions between conscious and unconscious processes (13). Dual-process theories posit that human behavior is governed by two systems: a reflective System 2 based on conscious deliberation and an impulsive System 1 driven by automatic, cue-triggered associative processes that lead to unconscious actions (14, 15). Initially, habitual behaviors may be driven by conscious intentions, such as self-efficacy. Over time, repeated actions in stable environments, reinforced by consistent cues, become automatic habits independent of ongoing motivation or intention (8). Studies confirm that established habits enable continued diabetes self-management behaviors, such as medication adherence, diet regulation, and exercise, through unconscious processes (16, 17). Therefore, based on dual-process theory, we hypothesize that habit strength may partially explain the association between self-efficacy and self-management behaviors.

Self-efficacy, defined as an individual’s confidence in their ability to consciously strive toward achieving goals, significantly influences perceptions and responses to tasks (18). Diabetes self-efficacy specifically refers to a patient’s belief in their capacity to successfully carry out diabetes management behaviors, leading to favorable disease management outcomes (19). Diabetes self-efficacy primarily operates through conscious cognitive mechanisms and has been identified as a key predictor of self-management behaviors (18). Previous studies (19) have identified self-efficacy as a core determinant in promoting and maintaining self-management behaviors among individuals with T2DM. However, the potential mechanisms through which diabetes self-efficacy is associated with self-management behaviors via habit strength remain unclear. In particular, limited research has examined how self-efficacy may contribute to behavioral engagement through habit formation. Therefore, further investigation of the relationships among these variables is warranted.

Based on the dual-process theory, individual behaviors are driven by both conscious control and unconscious automatic processes. Our previous studies have confirmed that conscious self-efficacy is a key predictor of self-management behaviors in patients with T2DM; concurrently, qualitative research has further elucidated the critical role of unconscious environmental factors in sustaining long-term self-management behaviors (19, 20). However, existing literature has yet to adequately quantify the specific mediating mechanism of unconscious habit strength between self-efficacy and self-management behaviors among patients with T2DM.

Accordingly, this study aims to: (1) assess the current level of self-management behaviors among patients with T2DM; (2) examine the relationships among habit strength, self-efficacy, and self-management behaviors; and (3) explore whether habit strength may serve as a mediator of the association between self-efficacy and self-management behaviors.

## 2 Methods

### 2.1 Study design and participants

This study is a multicenter, cross-sectional investigation that utilized convenience sampling to recruit participants from the endocrinology departments of four hospitals in Hainan Province, China. Patients were first screened by physicians according to the predefined inclusion and exclusion criteria and, if eligible, referred to trained researchers. Researchers conducted a secondary eligibility check (medical-record review) and then obtained written informed consent before investigation.

Inclusion criteria of the study were: (1) patients with T2DM who meet the diagnostic criteria outlined in the Chinese Guidelines for the Prevention and Treatment of Type 2 Diabetes (2020 edition); (2) aged between 18 and 65 years; (3) possessing normal cognitive and communication abilities, and are capable of independently completing the questionnaire or doing so with researcher assistance; (4) participants have provided informed consent and voluntarily agreed to take part in the study. Exclusion criteria were: (1) presence of psychiatric disorders; (2) severe diabetes-related complications (such as proliferative retinopathy, stage IV or higher nephropathy, or creatinine >2 mg/dL or 177 μmol/L, NYHA class III or higher heart failure, Wagner grade 1 or higher diabetic foot, etc.); (3) patients with cancer who have undergone radiotherapy or chemotherapy within the past 6 months.

The sample size was calculated using GPower 3.1, based on recommendations by Hair et al. (21). With an effect size of 0.15, a significance level of 0.05, and a statistical power of 0.80, the number of predictor variables was set at 2, yielding a sample size of 68. To meet the minimum sample size requirement for structural equation modeling, a final size of 200 was selected (22). Considering a 20% attrition rate and invalid questionnaires and attrition, the final target minimum sample size was set at 250.

### 2.2 Indicators and instruments

#### 2.2.1 Habit strength

The internationally recognized the Self-Report Habit Index (SRHI) was used for assessing habit strength (23). This scale provides standardized descriptive items that can be tailored to specific or general contexts, such as “the target behavior is something I do automatically”. In this study, five self-management behaviors of diet management, exercise, self-monitoring, foot care, and medication adherence were assessed, with wording adjustments as needed. After contextual adaptation and expert evaluation, a 20-item scale was adapted to assess the five above-mentioned behaviors, with each behavior evaluated by four items. The scale employs a five-point Likert scale, with 1 representing “completely disagree” and 5 representing “completely agree.” The total score ranges from 20 to 100, with higher scores indicating stronger habit strength (24). In the current study, the adapted SRHI demonstrated excellent internal consistency reliability (Cronbach’s *α* = 0.938). Exploratory factor analysis further supported its construct validity (KMO = 0.892; Bartlett’s *χ*^2^ = 8110.162, df = 190, *p* < 0.001), identifying five factors corresponding to the intended behavioral domains (diet management, exercise, self-monitoring, foot care, and medication adherence). These factors explained 89.88% of the total variance, with item communalities ranging from 0.742 to 0.961.

#### 2.2.2 Diabetes self-efficacy

The Chinese version of the self-efficacy for diabetes (SED) scale was used to measure diabetes self-efficacy (25). The scale comprises 9 items across four dimensions: diet, exercise, blood sugar management, and illness control. It employs a five-point Likert scale, where “1” signifies no confidence at all and “5” signifies complete confidence. The average score is calculated on a scale from 1 to 5, with higher average scores indicating greater self-efficacy. In this study, the Cronbach’s *α* coefficient was 0.833, indicating good internal consistency and reliability.

#### 2.2.3 Diabetes self-management behaviors

The Chinese version of the summary of diabetes self-care activities (SDSCA) scale was used to assess diabetes self-management behaviors (26). The scale consists of six dimensions: general diet (2 items), specific diet (2 items), exercise (2 items), blood sugar monitoring (2 items), foot care (2 items), and medication (1 item), totaling 11 items. The scale uses a Likert-like scoring system to reflect the number of days per week the patient engages in self-management behaviors, with ratings ranging from 0 to 7. The total score can range from 0 to 77. The fourth item is reverse-scored, while the remaining items are positively scored. Higher scores indicate better diabetes self-management behaviors. According to a standardized scoring approach reported in a previous study (27), the level of diabetes self-management behavior was categorized based on a percentage score calculated as: standardized score = (observed score / maximum possible score) × 100%. A standardized score of ≤40% was classified as poor, 41–80% as moderate, and >80% as good. In the present study, the Cronbach’s *α* coefficient of the SDSCA was 0.776.

#### 2.2.4 Participants’ characteristics

This instrument, developed by the researchers through a literature review, collects demographic information including age, gender, education background, marital status, employment status, individual monthly income, body mass index (BMI), duration of diabetes, duration of medication use, cohabitation group, the presence of complications, and study hospitals.

### 2.3 Data collection

After comprehensive training, five researchers conducted face-to-face data collection from March to July 2024 in the outpatient clinics and wards of four hospitals in Haikou City. Participants registered personal accounts on the Nanhai Diabetes Management App using their mobile phones and completed the online questionnaires through the “Self-Assessment” module. The Nanhai Diabetes Management App is an online diabetes management platform previously developed by our research team, which supports questionnaire reminders, data storage, and data export. In the present study, the app was used solely for questionnaire completion and data collection. The data collection procedure was as follows. First, researchers provided face-to-face technical guidance on account registration and app operation and instructed participants on how to access the “Self-Assessment” module. Second, participants completed and submitted the questionnaires independently through the app. After submission, researchers reviewed the questionnaires for missing responses and, when necessary, asked participants to complete any omitted items. Throughout the process, researchers did not participate in questionnaire completion, provide guidance regarding responses, or influence participants’ answers in any way. Data verification was independently performed by two researchers. The researcher-assisted technical support minimized missing data related to difficulties in operating the digital platform. A total of 292 questionnaires were distributed, and all 292 responses were valid, resulting in an effective response rate of 100%.

### 2.4 Ethical issues

The study was approved by the Ethics Committee of Hainan Medical University (Approval No. HYLL-2023-461). The study adhered to the principles outlined in the Declaration of Helsinki. Before the study began, participants were informed about the purpose and significance of the research. They were asked to provide written informed consent with full understanding before participating in the survey. Participants were also informed that they could withdraw from the study at any time without affecting their medical care. Upon completion, the research team securely stored the data to ensure confidentiality and anonymity.

### 2.5 Data analysis

Statistical analyses were performed using SPSS version 27.0, with a two-tailed significance level set at *p* < 0.05. Normality of continuous variables was assessed using the Kolmogorov–Smirnov test and by examining skewness and kurtosis values, with absolute skewness < 2 and kurtosis < 7 (28) considered indicative of approximate normality. Normally distributed data were expressed as mean ± standard deviation (SD), whereas non-normally distributed data were reported as median and interquartile range (IQR). Categorical variables were summarized as frequencies and percentages. Given that participants were recruited from four different study hospitals, the intraclass correlation coefficient (ICC) was calculated to evaluate potential clustering effects attributable to study hospitals.

Differences in diabetes self-management behavior scores across demographic and clinical subgroups were examined using independent-samples t tests or one-way analysis of variance for normally distributed data, and non-parametric tests for data that did not meet normality assumptions. Pearson correlation analysis was conducted to examine associations among self-efficacy, habit strength, and diabetes self-management behaviors.

Path analysis was conducted using the PROCESS macro (version 4.1) developed by Hayes, applying Model 4 to examine the potential mediating role of habit strength in the association between diabetes self-efficacy and diabetes self-management behaviors. Diabetes duration and study hospitals were included as covariates in the primary model to control for potential confounding effects. A bias-corrected bootstrap procedure with 5,000 resamples was used to estimate 95% confidence intervals (CI) for the indirect effect. Indirect effects were considered statistically significant when the 95% CI did not include zero. To evaluate the robustness of the findings, a sensitivity analysis was performed using a fully adjusted model. In addition to diabetes duration and study hospital, the model further adjusted for age, gender, marital status, education background, employment status, cohabitation group, BMI, individual monthly income, diabetes complication, medication duration.

## 3 Results

### 3.1 Participants’ characteristics

A total of 292 participants were recruited from four independent study hospitals, with 143, 76, 42, and 31 participants enrolled from each hospital, respectively. The intraclass correlation coefficient (ICC) for diabetes self-management behavior was 0.236, indicating that 23.6% of the variance in the outcome was attributable to between-hospital differences. Therefore, study hospital was included as a covariate in subsequent analyses to account for potential clustering effects. The mean age of the 292 patients with T2DM was 49.18 ± 10.04 years. Of the participants, 193 (66.1%) were male, 251 (86.0%) were married, 212 (72.6%) had an educational level below college, 210 (71.9%) were employed, and 207 (70.9%) lived with their spouses. Additional demographic and clinical characteristics are presented in Table 1. Among the baseline characteristics examined, diabetes duration and study hospitals were significantly associated with higher levels of self-management behaviors, whereas no significant associations were observed for other baseline demographic or clinical variables (Table 1).

**Table 1:** Demographic characteristics of the study participants and differences in self-management behaviors (*N* = 292).

Variable	*n* (%) *M* ± SD	Diabetes self-management behaviors		
		*M* ± SD	*t*/*F*	*p*
Age (years)	49.18 ± 10.04		−1.339	0.182
<45	81 (27.7)	32.44 ± 13.92		
≥45	211 (72.3)	34.81 ± 13.33		
Gender			0.127	0.899
Male	193 (66.1)	34.22 ± 13.37		
Female	99 (33.9)	34.01 ± 13.85		
Marital status			0.277	0.842
Unmarried	33 (11.3)	33.79 ± 10.11		
Married	251 (86.0)	34.09 ± 13.95		
Divorced	6 (2.0)	36.17 ± 10.05		
Widowed	2 (0.7)	42.00 ± 24.04		
Education background			1.519	0.221
Below primary level	54 (18.5)	31.33 ± 12.71		
Secondary and Post-secondary	158 (54.1)	34.55 ± 12.88		
College and above	80 (27.4)	35.26 ± 15.08		
Employment status			−0.516	0.606
Employed	210 (71.9)	33.90 ± 13.40		
Retired	82 (28.1)	34.80 ± 13.86		
Cohabitation group			1.417	0.238
Live alone	21 (7.2)	28.86 ± 11.257		
Parents living together	17 (5.8)	35.76 ± 10.47		
Spouses living together	207 (70.9)	34.80 ± 13.63		
Children living together	47 (16.1)	33.09 ± 14.59		
BMI			0.571	0.568
<24	146 (50.0)	34.60 ± 13.72		
≥24	146 (50.0)	33.70 ± 13.34		
Individual monthly income (Yuan)			0.796	0.452
<2000	113 (38.7)	32.90 ± 12.72		
2000–5,000	129 (44.2)	35.03 ± 13.29		
>5,000	50 (17.1)	34.70 ± 15.72		
Diabetes complication			3.616	0.058
Yes	87 (29.8)	36.45 ± 11.68		
No	205 (70.2)	33.18 ± 14.13		
Diabetes duration (years)	3.94 ± 4.40		−2.278	0.023
<3	183 (62.7)	32.77 ± 14.04		
≥3	109 (37.3)	36.47 ± 12.30		
Medication duration (years)	3.37 ± 4.32		−1.032	0.303
<2	182 (62.3)	33.52 ± 13.68		
≥2	110 (37.7)	35.20 ± 13.22		
Study hospitals			19.746	<0.01
Hospital 1	143	29.80 ± 13.34		
Hospital 2	76	37.78 ± 9.06		
Hospital 3	42	45.02 ± 13.28		
Hospital 4	31	30.61 ± 13.42		

BMI indicates Body Mass Index.

### 3.2 Scores of core variables

Skewness and kurtosis values for all measures were within acceptable ranges (absolute skewness < 2 and kurtosis < 7) (26), indicating that the data approximated a normal distribution (Table 2). Among the 292 participants, the mean total score for diabetes self-management behaviors was 34.15 ± 13.51, with a mean item score of 3.10 ± 1.22. The mean score corresponded to 44.4% of the maximum possible score and was classified as a moderate level of diabetes self-management behavior according to the standardized scoring criterion used in this study. The mean total score for self-efficacy was 24.98 ± 5.45, with a mean item score of 2.78 ± 0.61, reflecting a moderate level of self-efficacy. Similarly, the mean total score for habit strength was 56.77 ± 12.80, corresponding to a mean item score of 2.84 ± 0.64, suggesting a moderate level of habitual behavior (Table 2).

**Table 2:** Variable correlation analysis (*N* = 292).

Variables	Self-management behaviors	Self-efficacy	Habit strength
Total score (*M* ± SD)	34.15 ± 13.51	24.98 ± 5.45	56.77 ± 12.80
Mean Item score (*M* ± SD)	3.10 ± 1.22	2.78 ± 0.61	2.84 ± 0.64
Kurtosis	3.230	4.969	4.610
Skewness	−0.029	0.673	0.681
Self-management behaviors	1.000		
Self-efficacy	0.617^**^	1.000	
Habit strength	0.511^**^	0.677^**^	1.000

* indicates *p* < 0.05, ** indicates *p* < 0.01.

### 3.3 Variable correlation analysis

Pearson correlation analyses were conducted to examine the associations among habit strength, self-efficacy, and diabetes self-management behaviors. The total score of diabetes self-management behaviors was positively correlated with diabetes self-efficacy (*r* = 0.617, *p* < 0.01) and habit strength (*r* = 0.511, *p* < 0.01). In addition, diabetes self-efficacy was positively correlated with habit strength (*r* = 0.677, *p* < 0.01) (Table 2). These significant correlations provided preliminary support for the subsequent path analysis.

### 3.4 Role of habit strength in the association between self-efficacy and self-management behaviors

**Figure 1:**
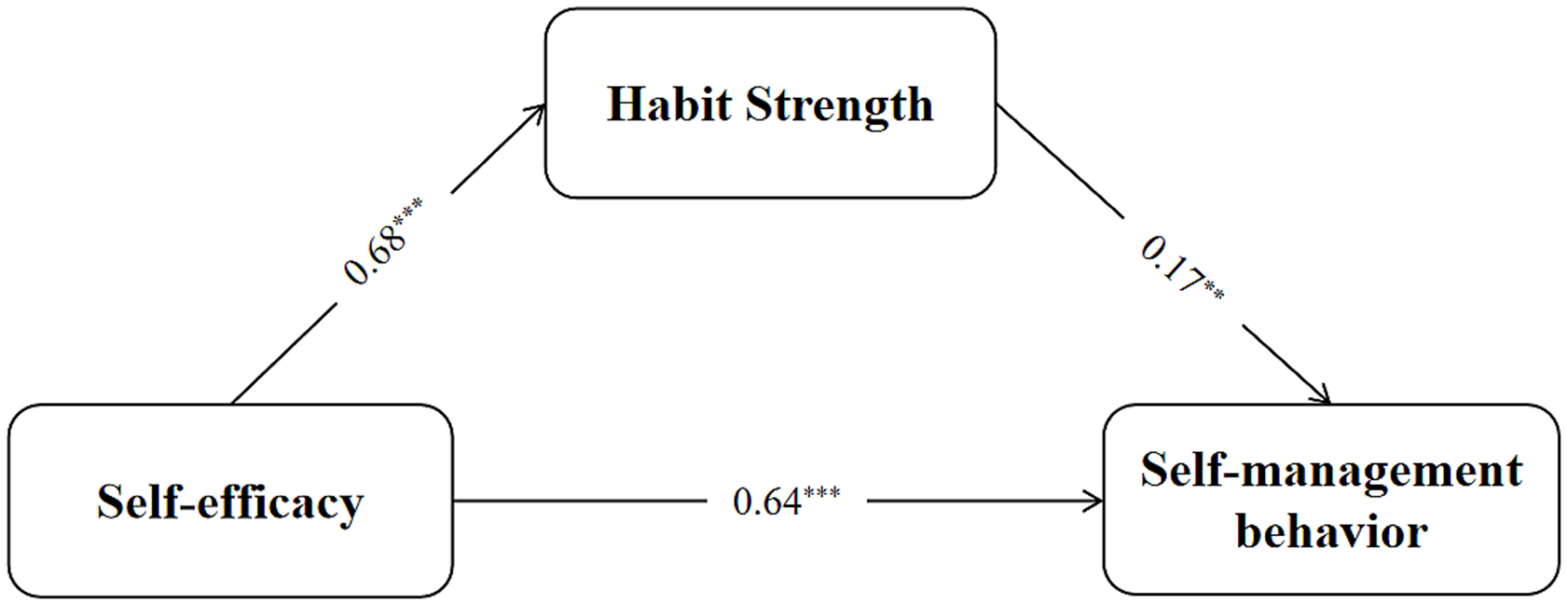
Path model illustrating the associations among self-efficacy, habit strength, and self-management behaviors. Values represent standardized path coefficients. Covariates included diabetes duration and study hospital. **p < 0.01, ***p < 0.001.

After controlling for duration of diabetes and study hospitals, path analysis showed that all estimated paths in the model were statistically significant (*p* < 0.001) (Figure 1). Self-efficacy was positively associated with diabetes self-management behaviors (*β* = 0.64, *p* < 0.001, 95% CI [0.54, 0.74]). After including habit strength in the model, the association between self-efficacy and diabetes self-management behaviors remained significant (*β* = 0.52, *p* < 0.001, 95% CI [0.40, 0.64]) (Table 3).

**Table 3:** Path analysis examining the associations among self-efficacy, habit strength, and self-management behaviors in patients with T2DM (*N* = 292).

Variable		*X* → *M*		*X* → *Y*		*X* → *M* → *Y*	
		β (95% CI)	*p*	β (95% CI)	*p*	β (95% CI)	*p*
Independent variable (Y)	Self-efficacy (X)	0.68 (0.60 ~ 0.76)	<0.001	0.64 (0.54 ~ 0.74)	<0.001	0.52 (0.40 ~ 0.64)	<0.001
Mediator variable	Habit strength (M)	−	−	−	−	0.17(0.05 ~ 0.29)	0.005
Control variable	Diabetes duration (years)	−0.02 (−0.10 ~ 0.06)	0.589	0.02 (−0.08 ~ 0.12)	0.528	0.02 (−0.06 ~ 0.10)	0.622
	Study hospitals	−0.00 (−0.04 ~ 0.04)	0.935	−0.07 (−0.15 ~ 0.01)	0.175	−0.07 (−0.15 ~ 0.01)	0.174
Model fit	*R^2^*	0.46	−	0.39	−	0.40	−
	Adjusted *R*^2^	0.45	−	0.38	−	0.39	−
	*F*	81.27***	−	60.21***	−	48.21***	−

Standardized β coefficients reported, and estimates were adjusted for diabetes duration and study hospitals; *X* indicates self-efficacy; *M* indicates habit strength; *Y* indicates self-management behavior; − indicates variable not entered at that step; ^*^ indicates *p* < 0.05, ^**^ indicates *p* < 0.01, ^***^ indicates *p* < 0.001.

In addition, self-efficacy was positively associated with habit strength (*β* = 0.68, *p* < 0.001, 95% CI [0.60, 0.76]), and habit strength was positively associated with diabetes self-management behaviors (*β* = 0.17, *p* < 0.01, 95% CI [0.05, 0.29]) (Table 3). The indirect effect was statistically significant, suggesting that habit strength may partially explain the association between self-efficacy and diabetes self-management behaviors.

### 3.5 Path analysis and effect decomposition

Bootstrap analyses showed that the 95% CI for both the direct effect of self-efficacy on diabetes self-management behaviors and the indirect effect via habit strength did not include zero (Table 4). The total effect of self-efficacy on diabetes self-management behaviors was *β* = 0.64 (95% CI [0.54, 0.74]), and the indirect effect through habit strength was *β* = 0.12 (95% CI [0.03, 0.22]). The indirect effect accounted for 18.6% of the total effect, suggesting that habit strength may partially explain the association between self-efficacy and diabetes self-management behaviors.

**Table 4:** Bootstrap results for direct and indirect effects in the path analysis (*N* = 292).

Effect type	*β*	SE	95% CI		Effect size (%)
Total effect	0.64	0.05	0.54	0.74	100%
Direct effect	0.52	0.08	0.39	0.65	81.4%
Indirect effect	0.12	0.05	0.03	0.22	18.6%

Standardized *β* coefficient reported, and estimates were adjusted for diabetes duration and study hospitals.

### 3.6 Sensitivity analysis

To assess the robustness of the findings, a fully adjusted model was fitted by additionally controlling for age, gender, marital status, education background, employment status, cohabitation group, BMI, individual monthly income, diabetes complication, diabetes duration, medication duration, study hospitals. The results were consistent with those of the primary analysis. Self-efficacy remained positively associated with diabetes self-management behaviors (*β* = 0.66, *p* < 0.001, 95% CI [0.55, 0.76]). After habit strength was included in the model, the association between self-efficacy and diabetes self-management behaviors remained significant (*β* = 0.54, *p* < 0.001, 95% CI [0.41, 0.67]). These findings suggest that the mediation model was robust after adjustment for additional potential confounding variables (Table 5).

**Table 5:** Sensitivity analysis of the path model using a fully adjusted model among patients with T2DM (*N* = 292).

Variable		*X* → *M*		*X* → *Y*		*X* → *M* → *Y*	
		*β* (95% CI)	*p*	*β* (95% CI)	*p*	*β* (95% CI)	*p*
Independent variable (Y)	Self-efficacy (X)	0.66 (0.57 ~ 0.76)	<0.001	0.66(0.55, 0.76)	<0.001	0.54(0.41 ~ 0.67)	<0.001
Mediator variable	Habit strength (M)	−	−	−	−	0.17 (0.05 ~ 0.30)	0.007
Control variable	Age (years)	0.05 (−0.05 ~ 0.15)	0.356	0.03 (−0.07 ~ 0.12)	0.630	0.02 (−0.08 ~ 0.12)	0.736
	Gender	−0.04 (−0.14 ~ 0.06)	0.375	−0.00 (−0.10 ~ 0.09)	0.930	0.00 (−0.09 ~ 0.10)	0.956
	Marital status	−0.02 (−0.12 ~ 0.07)	0.611	−0.03 (−0.13 ~ 0.06)	0.493	−0.03 (−0.13 ~ 0.07)	0.542
	Education background	0.06 (−0.04 ~ 0.16)	0.232	−0.00 (−0.10 ~ 0.09)	0.929	−0.02 (−0.11 ~ 0.08)	0.777
	Employment status	−0.06 (−0.16 ~ 0.04)	0.231	0.07 (−0.03 ~ 0.16)	0.218	0.08 (−0.02 ~ 0.17)	0.151
	Cohabitation group	0.09 (−0.01 ~ 0.19)	0.051	0.05 (−0.05 ~ 0.15)	0.287	0.04 (−0.06 ~ 0.13)	0.450
	BMI	−0.10 (−0.20 ~ −0.00)	0.029	−0.01 (−0.10 ~ 0.09)	0.912	0.01 (−0.09 ~ 0.11)	0.809
	Individual monthly income (Yuan)	0.03 (−0.07 ~ 0.12)	0.627	0.07 (−0.03 ~ 0.17)	0.202	0.07 (−0.03 ~ 0.16)	0.225
	Diabetes complication	0.02 (−0.08 ~ 0.12)	0.629	0.14 (0.04 ~ 0.24)	0.006	0.14 (0.04 ~ 0.23)	0.006
	Diabetes duration (years)	0.00 (−0.10 ~ 0.10)	0.985	−0.02 (−0.11 ~ 0.08)	0.798	−0.02 (−0.11 ~ 0.08)	0.793
	Medication duration (years)	−0.05 (−0.15 ~ 0.05)	0.441	−0.03 (−0.12 ~ 0.07)	0.689	−0.02 (−0.12 ~ 0.08)	0.780
	Study hospitals	0.00 (−0.10 ~ 0.10)	0.956	−0.07 (−0.17 ~ 0.03)	0.160	−0.07 (−0.17 ~ 0.03)	0.153
Model Fit	*R* ^2^	0.49	−	0.41	−	0.42	−
	Adjusted *R*^2^	0.46	−	0.38	−	0.40	−
	*F*	22.13***	−	16.15***	−	15.80***	−

The fully adjusted model controlled for age, gender, marital status, education background, employment status, cohabitation group, BMI, individual monthly income, diabetes complication, diabetes duration, medication duration, study hospitals; *M* indicates habit strength; *Y* indicates self-management behavior; Standardized β coeffcients reported; *X* indicates self-efficacy; − indicates variable not entered at that step; ^*^ indicates *p* < 0.05, ^**^ indicates *p* < 0.01, ^***^ indicates *p* < 0.001.

## 4 Discussion

This multicenter cross-sectional study examined the current levels of self-management behaviors among patients with T2DM and explored the relationships among habit strength, self-efficacy, and self-management behaviors, as well as the potential role of habit strength. The findings suggest that self-management behaviors among patients with T2DM remain moderate. Both self-efficacy and habit strength were positively associated with self-management behaviors, and habit strength may partially explain the association between self-efficacy and self-management behaviors.

Our findings reveal that the self-management behavior scores of T2DM patients were lower than those reported by Zhang et al. (29). A possible reason is that over 62.7% of the T2DM patients included in this study had a disease duration of less than 3 years. Patients in this early stage of the disease often have limited awareness of diabetes-related knowledge. Therefore, through leveraging specialized healthcare platforms to strengthen stepwise health education for newly diagnosed patients and implement personalized intervention programs, tangible and targeted support can be effectively provided for the comprehensive management of diabetic patients.

This study found that habit strength is positively correlated with diabetes self-management behaviors, which is consistent with the research by Cummings et al. (30) and Gardner (10). Several factors may explain this: First, high habit strength suggests that behaviors are more unconscious and automatic, facilitating adherence to health management practices, such as timely medication intake and blood glucose monitoring, without requiring extensive deliberation (30). Second, patients with strong habits are more likely to sustain self-management behaviors in the long term, leading to stabilized blood glucose levels. Finally, the consistent practice of self-management behaviors yields positive health outcomes, which further reinforce habit formation (10). Recent studies indicate that habits are behavioral patterns learned through context-dependent repetition (10, 31). Repeated execution of behaviors in a consistent environment strengthens the association between the context and the behavior, to the extent that encountering the context alone can automatically trigger the habitual response (32). Therefore, healthcare professionals can design interventions that utilize environmental cues to promote the repeated execution of behaviors such as blood glucose monitoring and proper medication adherence, thereby supporting the development of effective diabetes self-management habits.

The study also demonstrates that self-efficacy in T2DM patients is positively correlated with self-management behaviors, suggesting that higher self-efficacy is associated with better self-management. This finding aligns with previous research (19, 33). The underlying rationale is that patients with high self-efficacy are more proactive in addressing health issues, make informed health decisions, and believe in their ability to manage their condition effectively. Previous research by Jiang et al. (19) confirmed that a self-efficacy-based structured education program can promote self-management behaviors in patients with T2DM by enhancing their self-efficacy. They are more likely to actively seek information on blood glucose management, consider the effects of diet and exercise on blood glucose, and ultimately exhibit superior self-management behaviors (34).

Additionally, it is noteworthy that habit strength mediated the relationship between self-efficacy and diabetes self-management behaviors, with the indirect effect accounting for 18.6% of the total effect. Although this mediation effect was of a small-to-moderate magnitude, the findings suggest that self-efficacy may promote diabetes self-management behaviors partly through strengthening habit formation, accounting for approximately one-fifth of the overall effect. The consistency of the findings in the fully adjusted sensitivity analysis further supports the robustness of this mediation pathway. This finding suggests that, in addition to enhancing self-efficacy, diabetes self-management interventions may benefit from incorporating habit-based strategies aimed at strengthening the automaticity and sustainability of self-management behaviors. In relatively stable environments, diabetes self-management behaviors may initially be supported by reflective processes, such as self-efficacy, which facilitate individuals’ willingness to invest effort in managing their condition (35). Over time, repeated behavioral performance in consistent contexts may contribute to the development of habit strength, whereby environmental cues can prompt behavioral responses more automatically (9, 11). As habit strength increases, behaviors may become less reliant on conscious regulation and more readily triggered by recurring contextual cues (36).

Our study initially reveals that self-management behaviors in patients with T2DM are shaped by both conscious self-efficacy and unconscious habit strength. This perspective highlights the potential importance of habit formation in sustaining health behaviors. Prior research has suggested that self-efficacy alone may not be sufficient to maintain long-term self-management, as consciously regulated behaviors can be disrupted by contextual and individual factors, such as variations in disease awareness or management experience (20). In contrast, habitual behaviors may provide more stable support for sustained engagement by reducing cognitive demands and enabling more automatic behavioral responses (9, 37). Consistent with this view, emerging evidence indicates that interventions targeting habit formation may help improve self-management behaviors in individuals with diabetes (17). These findings are broadly consistent with dual-process perspectives, which emphasize the complementary roles of reflective and automatic processes in shaping health behaviors.

Therefore, when developing self-management support strategies for individuals with T2DM, healthcare professionals and researchers may consider not only strengthening self-efficacy but also supporting habit formation. Structured education programs can be used to enhance self-efficacy by promoting patients’ confidence and engagement in self-management. At the same time, approaches that facilitate habit formation may be incorporated, such as the use of environmental cues and digital tools that provide reminders, prompts, and ongoing feedback to support consistent behavioral performance. Together, these strategies may help reduce cognitive burden and support more sustained engagement in self-management behaviors among individuals with T2DM (38, 39).

### 4.1 Strengths and limitations

This multicenter cross-sectional study included participants from four hospitals in Hainan Province, China, which may enhance the representativeness of the sample within the regional context. The study provides evidence on the associations among self-efficacy, habit strength, and self-management behaviors in individuals with T2DM. In particular, the findings suggest that habit strength may help explain the association between self-efficacy and self-management behaviors, offering empirical support for perspectives that consider both reflective and more automatic processes in health behavior regulation. These findings may inform the development of interventions that integrate both motivational and habit-based approaches to support diabetes self-management.

Despite these strengths, several limitations should be considered. First, the sample was drawn from a single province in China (Hainan), China’s only tropical island with unique climate and dietary habits. While the findings provide valuable insights within this specific context, their generalizability to other geographic regions and populations may be limited. Future studies should conduct multicenter surveys across diverse geographic areas to enhance external validity. Second, all variables were assessed using self-reported measures, which may be subject to reporting bias. In addition, the study did not include objective clinical indicators (e.g., HbA1c) to validate self-reported behaviors and outcomes. Future research should incorporate objective measures and multi-method assessment approaches to strengthen the validity of the findings. Third, although participants completed the questionnaires independently, the presence of researchers during data collection may have introduced social desirability bias and could partly explain the exceptionally high valid response rate (100%). In addition, the researcher-assisted administration process may have resulted in potential selection bias. Future studies should consider fully anonymous self-administered surveys to further reduce these potential biases and validate the robustness of the findings. Finally, due to the cross-sectional design, causal inferences cannot be established, and the observed associations should be interpreted with caution. Longitudinal or experimental studies are needed to further examine the temporal and potential causal relationships among these variables.

## 5 Conclusion

This study found that self-management behaviors among patients with T2DM were moderate. Habit strength was positively associated with self-management behaviors and may help explain the association between self-efficacy and self-management behaviors. These findings provide empirical support for perspectives that consider both reflective and more automatic processes in shaping health behaviors. Both self-efficacy and habit strength may represent relevant targets for future diabetes self-management interventions. Healthcare professionals may consider strategies that enhance patients’ confidence and engagement while also supporting the development of consistent behavioral patterns. For example, structured education programs can be combined with approaches that facilitate habit formation, such as the use of environmental cues and digital tools to provide reminders, prompts, and ongoing feedback. Such integrated strategies may help support more sustained self-management behaviors. Future longitudinal studies and randomized controlled trials are needed to further examine these associations and evaluate the effectiveness of combined intervention approaches.

## Data Availability

The raw data supporting the conclusions of this article will be made available by the authors, without undue reservation.
